# Improving Science Education for Sustainable Development

**DOI:** 10.1371/journal.pbio.0050306

**Published:** 2007-12-11

**Authors:** Michiel van Eijck, Wolff-Michael Roth

## Abstract

Data from an environmental education project demonstrate how a learning framework that is consistent with contemporary ethology, and represents humans as self-determined yet integral parts of their environment, contributes both to the improvement of education and to a sustainable future.

In recent issues of noteworthy journals, natural scientists have argued for the improvement of science education [1–4]. Such pleas reflect the growing awareness that high-quality science education is required not only for sustaining a lively scientific community that is able to address global problems like global warming and pandemics, but also to bring about and maintain a high level of scientific literacy in the general population. There is no doubt that effective education can serve as a vehicle for solving global problems. The problem centers on how to achieve more effective education.

We believe that science education would greatly benefit from incorporating the lessons of cognitive science and contemporary ethology to provide a framework for explaining human behavior grounded in evolutionary theory. According to such a perspective, humans collectively produce and reproduce their environment through their actions and are therefore capable of acting responsibly for a sustainable future.

In order to design education that will effectively contribute to a sustainable future, we argue for a learning framework that is consistent with contemporary ethology and represents human beings as self-determined yet integral parts of their environment. Such an epistemology is more holistic than traditional and often reductionistic frameworks in education and draws on the central paradigm of the life sciences—evolution. Furthermore, this framework approaches collective human activity as the pivotal unit of analysis in which individuals transact with the social and natural world. Drawing on data from an environmental education project, we demonstrate how this epistemology allows us to contribute both to the improvement of education and to a sustainable future of life on earth.

## A Framework for Science Education

*Nothing in biology makes sense except in the light of evolution. [5]*

In the 1920s, Soviet psychologists—including Lev Vygotsky and his colleagues Alexander Luria and Aleksei Leont'ev—argued that “cognition does not exist outside the life process that in its very nature is a material, practical process. The reflection of reality arises and develops in the process of the development of real ties of cognitive people with the human world surrounding them; it is defined by these ties and, in its turn, has an effect on their development” [6]. To understand human cognition as a result of both evolutionary (historical) and cultural development integrated with its natural material environment, Vygotsky and his collaborators formulated a completely new psychological framework called cultural-historical activity theory. At the core of this framework is the notion that the individual never directly acts in or reacts to (with inborn reflexes) the environment, but that objects or artifacts, such as tools, mediate the relationship between the human subject and objects of environment [7]. Tool use is not limited to humans but is a critical evolutionary step associated with higher-order cognitive processes common in animal species such as chimpanzees [8] and crows [9]. In thinking of human activity as a complex evolutionary achievement, its emergence may be conceptualized in three steps [10]. First, animal activity can be thought of as an immediately collective and populational “methodology of survival” of a species (Figure 1). This type of activity is not just passive—we should speak of construction of the environment rather than adaptation to the environment [11].

**Figure 1:**
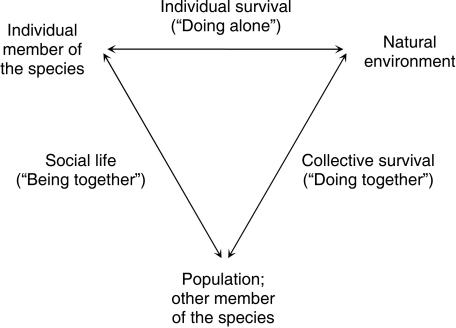
General Structure of the Animal Form of Activity after Engeström [10].

Second, in animal evolution, each of the three sides of the triangle depicted in Figure 1 thus exhibits evolutionary development. The emerging utilization of tools therefore evolves at the uppermost side of “individual survival.” Collective traditions, rituals, and rules, originating at the crossing of adaptation and mating, emerge as part of “social life” on the left-hand side; in meta-analytic studies, primatologists have reported such collective, cultural traditions among chimpanzees [12] and orangutans [13]. On the right-hand side, the “collective survival” exhibits an evolving division of labor, influenced by the practices of breeding, upbringing, and mating, and appearing first as the division of labor between the sexes (Figure 2).

**Figure 2:**
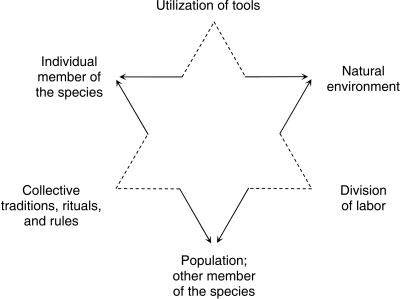
Structure of Activity in Transition from Animal to Human after Engeström [10].

Third, the emerging mediators on each side of the triangle depicted in Figure 2 become unified determining factors that mediate human activity. The ecological and natural become the economic and historical. In cultural-historical activity theory, then, activity is understood as some macrolevel formation that serves the survival of the collective—such as farming, education, or environmentalism [14]. The model of human activity thus allows us to understand such activities as a set of relations (Figure 3). Yet, in accordance with an evolutionary paradigm, the task is to grasp the systemic whole, not just to reduce human activity to its separate connections.

**Figure 3:**
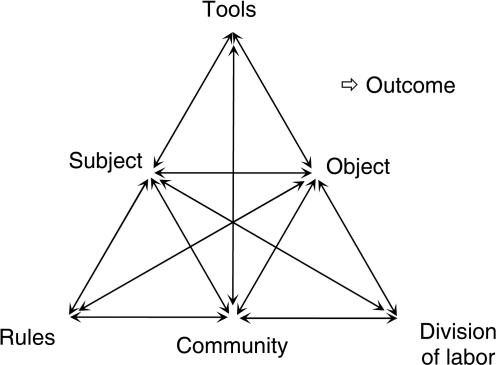
The Structure of Human Activity after Engeström [10].

Central in the model is the concept of object-oriented and artifact-mediated activity: artifacts are any tool, sign, or other means required for the mediation between the subject, a human agent, and the object, the thing or change in the environment with which a human interacts [7]. This interaction is mediated further by the other moments characteristic of human activity: rules, division of labor, and community (culture). The community comprises multiple individuals or subgroups who share the same general object and who distinguish themselves from other communities. The division of labor refers to both the horizontal division of tasks between the members of the community and to the vertical division of power and status. Finally, the rules refer to the explicit and implicit regulations, norms, and conventions that constrain actions and interactions within the activity system. In groups of humans, knowledge is a dynamic set of artifacts that simultaneously mediate and are produced by activity.

Cultural-historical activity theory allows us to better understand knowledge, which resides not only in the heads of individuals but also in activity itself. For example, in tool use, part of the knowledge in action resides in the tool. Knowledge is inferable from patterned actions, and actions leave traces in human bodies, which subsequently mediate actions in the future. Such traces can be conceptualized as artifacts. Accordingly, knowledge can be seen as part of object-oriented and artifact-mediated activity [7]. Importantly, sense and meaning are characteristic of activities as a whole rather than of actions in themselves. While tacit operations that constitute actions are embodied in individuals, they have their origin in mimetically copied or routinized culturally meaningful action and therefore constitute a crystallized form of social action.

**In order to design education that will effectively contribute to a sustainable future, we argue for a learning framework that is consistent with contemporary ethology and represents human beings as self-determined yet integral parts of their environment.**

We will demonstrate how cultural-historical activity theory allows us to design science education to build a sustainable future with a case study from the practice of environmentalism. Currently, schooling does not give students opportunities to participate in setting the goals and objects of their activities, choosing tools, determining the division of labor, or constructing the rules that shape how people interrelate with each other and their environment. As a result, students must perform like lab rats to reap benefits in the form of grades, points, stars. They engage in defensive learning to avoid punishment, as one of Leont'ev's intellectual students put it [15]. There are other ways to organize school science that provide students precisely with the kind of learning opportunities that characterize other everyday, science-related activities, including laboratory and field sciences or environmentalism. As they pursue their goals, students engage in whatever they deem necessary to expand their horizon of possible actions. The actual expansion of this horizon is a sign that they have learned.

## Effective Education for Sustainable Development: An Example

We have assisted local middle school teachers to implement a curriculum focusing on the watershed of one community in western Canada, allowing students to learn whatever science they needed (biology, environmental science, chemistry, physics) while studying the main creek draining the watershed. This curriculum embodied the lessons derived from teaching science over more than a decade, including the involvement of scientists, environmentalists, water technicians, farmers, aboriginal elders, community politicians, and parents. We taught the curriculum three times, each time improving on any problems found in the previous iteration(s). For example, during the second and third time we taught the course, we no longer required all students to collect correlational data and to produce graphs, because we found out during the first time that girls and aboriginal students especially were turned off by such tasks. Although students conducted their own research and presented it to others for more than 70% of the time, more traditional teaching methods constituted an integral part of the curriculum unit. For example: an environmentalist with a B.Sc in biology presented a slide-supported lecture on the concept of watershed; together with an invited scientist, the regular teacher organized a data analysis session in which small groups of students analyzed the same student-collected, pooled dataset; and students received direct instruction from a teacher, a scientist, or a fellow student in using standard scientific tools, such as dissolved oxygen meters, colorimeters for determining turbidity, or a microscope to study arthropods and other microorganisms. We will outline the salient details of this unfolding curriculum and point out its significance for science education. However, as we have committed ourselves to ethical guidelines for research with human subjects, we cannot provide all details that readers may find interesting because in some cases this would violate the ensured anonymity of the participants.

**Science educators have struggled for decades with the question of how to design and evaluate curricula through which scientific knowledge does not end up in isolated, artificial settings such as tests, but leaves sustainable traces in students' daily lives**.

We began the curriculum with a lesson in which the students read several articles from the local biweekly newspaper concerning (a) the health of the local watershed, (b) the watershed- and creek-focused actions of an environmentalist group, (c) the struggle of one citizen group to be connected to the water main that supplies water to all other residents, and (d) other water-related activities in the community (meetings, water advisory task force, etc.). In one of the articles, the director of the environmentalist group was interviewed: she not only described the sorry environmental health of the watershed and creek but also invited all community members to contribute to a better understanding of the watershed and revitalization of parts of the creek and watershed.

In all iterations, the seventh-grade students felt personally involved. Some of their parents were fishermen affected by the toxic run-offs from the creek around the inlet in which the community is located (the salmon species include chum [Oncorhynchus keta], coho [O. kisutch], and chinook [O. tshawytscha]), and others were from the local aboriginal band, which still drew sustenance from the oysters (Ostrea lurida, Crassostrea gigas), mussels, clams (Saxidomus giganteus, Tapes japonica, Clinocardium nuttalli), crabs (Cancer sp.), sea urchins (Strongylocentrotus sp.), and barnacles (Balunus sp.) found on the beaches near their village. At the time, 12 of 15 shellfish beaches were closed due to fecal contamination. Recognizing how creek pollution affected their lives and the life of their community, students immediately began a discussion concerning their involvement: how to help, what to study, where to report their findings, and so forth.

The following week—drawing on parent volunteers as drivers and environmentalists, university biology students, and scientists as experts—the students first visited the creek at various places. They discovered pristine parts of the valley (Figure 4), and learned from the elders, water technicians, or environmentalists that this watershed provided rich habitat and therefore food for the local aboriginals and original (19th century) settlers, including 24-inch cutthroat trout (O. clarki). But the students also discovered large parts of the creek that have been straightened and dredged to function as ditches for the rapid discharge of water from what had once been a wetland habitat that the local aboriginal tribes used as their hunting ground (Figure 5). They discovered the small industrial area at the end of the photograph (covered by the tall fir trees), where several companies discharge their effluents into the creekside arm leading to its high load of heavy metals, organic compounds, and other pollutants. The students also learned that in most reaches, the creek is no longer a viable habitat because the water flows too slowly, there is not enough oxygen, there is insufficient riparian vegetation to prevent the heating up of the creek, and so forth. During their visit to the mouth of the creek, the students found out that the contaminants (including sewage effluents, storm drainage, and agricultural runoff) from the creek, as reported in a water quality study, pollute the inlet and lead to “marine habitat disturbances,” “disturbances of the sensitive coastal ecology,” and “environmental degradation.”

**Figure 4:**
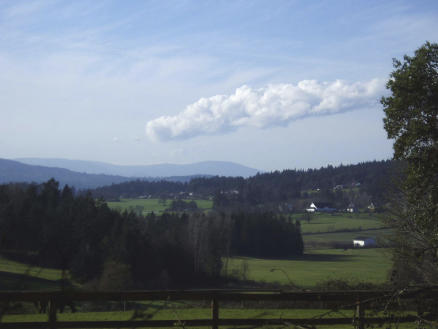
Pristine Parts of the Valley Some of the valley still constitutes pristine environment, which is mostly used for farming.

**Figure 5:**
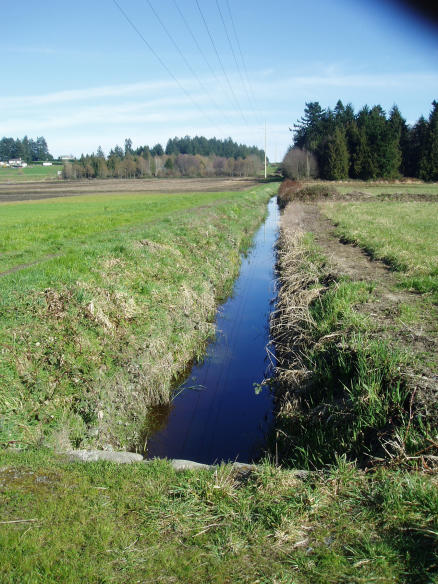
The Creek Many parts of the creek have been straightened to produce ditches that lead to faster run-off; an industrial park behind the tall trees to the right spills heavy metals and other pollutants into this part of the creek, affectionately called “stinky ditch.”

This visit really got the students excited: in the health of the creek and watershed, they had found an object (Figure 3) for their involvement in a local community issue, and, working in groups of three or four students, began to decide what they wanted to do and produce as an outcome (Figure 3). Because students decided upon what they wanted to produce, they felt in control; and in the course of pursing their research over the subsequent three to four months, they identified gaps in their knowledge that they needed to fill to achieve their objectives. They decided upon the division of labor (Figure 3) within their groups, drawing among others on equity as a rule to ensure that they all had opportunities for learning.

As students engaged in a variety of research projects, they changed their understanding and, correspondingly, evolved their research programs. Students chose to respond to the environmentalists' call for creating an understanding of and about the creek in different ways, each way corresponding to the particular learning needs of the students within a group. For example, one group of boys decided to determine, among other questions, whether there was a relationship between stream speed and the creek profile; in another project, students determined the relationship between the frequency of certain organisms (e.g., Arthropoda) and stream speed. Another group of girls decided to document creek health by means of verbal descriptions recorded on tape onsite and photographs showing the effects of pollution, pollutants (“garbage”), and the like (Figure 6). The girls also recorded their interviews with local politicians and community elders, which they transcribed for subsequent “publication” purposes.

**Figure 6:**
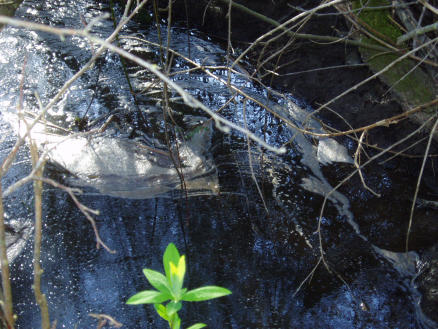
Documentation of Effluents Entering the Sidearm of the Creek, as Recorded by Seventh-Grade Students

Another group focused on the water itself: they used a dissolved oxygen meter and a colorimeter borrowed from the environmentalists to collect samples at various sites along the creek and studied the prevalence of organisms in the different sites. Finally, one particularly interested student, after having used a more qualitative test for the prevalence of fecal coliform bacteria, became so engaged that he enlisted the help of a graduate student involved and accessed a microbiology laboratory to produce more reliable estimates of the coliform counts. This allowed him to correlate high coliform loads with particular farms. In one instance, a student chose not to contribute to the creek studies at all, but then, during a brainstorming session with us, decided to become the “historian” of his class's effort, using one of our video cameras to document what students were doing and interviewing his peers about the projects they conducted, their aims and rationales, and the ultimate outcomes they wanted to produce. In all of these projects, the tools (Figure 3) mediated students' actions and therefore constituted one aspect that determined the high quality of the measurements produced.

Although the different groups pursued specific projects, students' learning extended beyond what they discovered through their own projects. We held regular discussions with the whole class, so that students in any one group could learn about what other groups found out, which tools they used, and how they used them. Sometimes the entire class got involved in analyzing the data generated by a specific group, such as when one group made available their incidence versus stream-speed data for 10 different species. The knowledge generated by individual groups therefore re-entered the classroom community (Figure 3), which “absorbed” or “consumed” it by drawing on it as a resource for subsequent investigations. In several instances, students from a class that had already completed the unit assisted in introducing first other teachers and then their peers from other classes to this unit, including data collection and other elements of the curriculum. These students also came to the field to serve as instructors for one or more groups while they conducted their studies in and around the creek; as instructors, they therefore participated in the division of labor (Figure 3) among the teachers and others facilitating the efforts of those currently going through the curriculum.

In the end, students reported the outcomes (Figure 3) of their work during the open-house event that the environmentalists organized each year. The students' work was spread throughout the room among other stations mounted by environmentalists and other community members (e.g., a heritage group, scientists from a nearby marine research station). Stations featured several items, such as a Web site (using a computer installed for the purpose), trays containing specimens and guides for classification to teach visitors about microorganisms, information on the use of dissolved oxygen meter and colorimeter, and mounted posters. Throughout the two-day events, the seventh-grade students interacted in knowledgeable ways with visitors of all ages. For example, our videotapes show one seventh-grade student explaining, using a physical model he had constructed, the inner workings of a watershed to a child several years younger (Figure 7A); another student explained to a university law professor how to measure the turbidity of water (Figure 7B); at a third station, a student presented the photographs, observation transcripts, and interviews with elders and politicians (Figure 7C); and at a fourth station, two students introduced several adults to the classification of arthropods and mayfly nymphs (Ephemeroptera) and explained how to distinguish larvae that look similar. All of these interactions between students and visitors are evidence of the tremendous knowledge that the students have gained through their participation.

**Figure 7:**
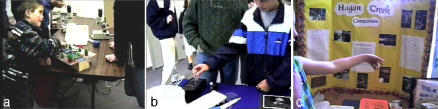
The Open-House Event These video offprints from a public open-house event organized by an environmentalist group feature moments where students helped others to learn about the creek.

But there was more to this curriculum unit than the learning of individual students. The students' products also were featured in the local newspaper and on the Web site of the environmentalist group; where applicable, measurements of oxygen, pH, and turbidity levels were entered into the group's databases, to which others (university students, residents, environmentalists) already had contributed data. The students' work, therefore, was re-entering the community at large (Figure 3), which, by consuming and absorbing these products, underwent sustainable change toward a more positive, environmentally healthy future for the watershed. But this re-entering of knowledge into the community also generated tensions, when, for example, students were not allowed back onto those farms where a student had found significant and reliably measured increases in coliform bacteria levels.

Without the various people and groups involved, this curriculum unit would not have unfolded in the ways it did during our three iterations. An important aspect was the contributions others made (parents, elders, politicians, scientists, environmentalists, graduate students). These contributions constitute a form of division of labor at the level of the community (Figure 3), where “education” no longer was held to be the prerogative of school and teachers but an issue for the community as a whole. And participation provided both those within and outside the school with a learning experience: students learned science, teachers learned how to teach such a unit, and the community learned about the environmental health of the watershed they inhabited.

## Lessons Learned

The curriculum described here, taught in three iterations, achieved its goal of raising scientific literacy generally rather than reaching but a few (gifted) students who would have been successful however science had been taught. By all measures, students not only learned science but learned more than they would have in normal science lessons. They also helped guide their community toward a sustainable future. These students did not merely acquire a stock of words to reproduce on a test, or copy notes that would be thrown away after the unit ended, but participated in changing their community. During the environmentalist open-house events, they also taught visitors of all ages scientific processes and contents. Most importantly, as our immediate and long-term (12 to 18 months) post-unit interviews with the students showed, they had been able to collect evidence that their participation in the environmental cause had brought about lasting changes—including the identification of heavy polluters. For example, one student noted:

*I worked very hard on the map and proceedings. During this course I learned about fieldwork: I learned how to collect samples of the creek and take temperatures and speed. I also did some work with the community. [This unit] taught me about working with others and working in the community. I noticed that ever since our Henderson Creek article was published in the Peninsula News Review that the public has begun to notice the creek.*

As a result of the efforts of the students and others, this community today is different from what it was prior to the first curriculum—e.g., the official community plan has been changed, fences have been built to protect riparian areas (Figure 8), artificial riffles now oxygenate parts of the creek, and the industrial pollution has decreased. The environmentalists attributed a considerable aspect of their success to the students' involvement, both through their public work and through the sensibilization and conscientization to environmental issues within their extended families [16]. That is, in this community, practical scientific literacy (rather than passive words in a person's mind) has increased, in particular with respect to the environment and environmental health.

**Figure 8:**
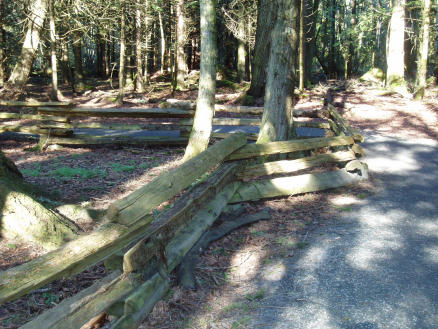
A Fence Protecting the Riparian Area Due to the environmental movement, to which three classes of seventh-grade students actively contributed, the community has changed both physically and in its attitudes toward the environment.

The theoretical model we propose here for rethinking science curricula is suited ideally for engendering actions toward sustainability, as it had been developed to bring about, support, and understand expansive learning, which occurs when people (workers, environmentalists, teachers) work together to change their environment for a better future [14]. Underlying expansive learning is the recognition that collectively we can achieve more and better control our environment and future than if we attempted to work individually. This future is better in part because the decisions and processes of change involve whole communities who, consistent with their democratic values, both envision and work toward a better life.

Science educators have struggled for decades with the question of how to design and evaluate curricula through which scientific knowledge does not end up in isolated, artificial settings such as tests, but leaves sustainable traces in students' daily lives [17]. According to our model, such traces cannot be seen independently from the activities in which students engaged and will engage in their future. Educational design and evaluation of environmental programs should therefore appropriate the cultural-historical aspects of human activity. The effect of such programs, then, should be measured as permanent and sustainable changes in the community brought about by students as human beings rather than as caged lab rats for whom a sustainable future only glooms out of reach in their artificial environment.
